# Population Genomic Survey of *Hypophthalmichthys molitrix* in the Yangtze River Basin: A RAD Sequencing Perspective

**DOI:** 10.3390/ani15192906

**Published:** 2025-10-05

**Authors:** Weitao Li, Xingkun Hu, Yanfu Que, Ezhou Wang, Nian Xu, Ke Shao, Guoqing Lu, Xiaolin Liao, Bin Zhu

**Affiliations:** 1 Key Laboratory of Ecological Impacts of Hydraulic-Projects and Restoration of Aquatic Ecosystem of Ministry of Water Resources, Wuhan 430079, China; liweitao10@163.com (W.L.); huxingkun@mail.ihe.ac.cn (X.H.); yfque@mail.ihe.ac.cn (Y.Q.); wezsstsyy@mail.ihe.ac.cn (E.W.); xunian@mail.ihe.ac.cn (N.X.); shaoke86@mail.ihe.ac.cn (K.S.); 2 Institute of Hydroecology, Ministry of Water Resources and Chinese Academy of Sciences, Wuhan 430079, China; 3 Department of Biology, University of Nebraska at Omaha, Omaha, NE 68137, USA; glu3@unomaha.edu; 4 Fisheries College, Hunan Agricultural University, Changsha 410128, China

**Keywords:** *Hypophthalmichthys molitrix*, genetic diversity, population structure, RAD sequencing, Yangtze River populations

## Abstract

**Simple Summary:**

This study analyzed genetic diversity and structure in silver carp (*Hypophthalmichthys molitrix*). Samples came from 17 Yangtze River sites plus one U.S. population. RAD-seq yielded 759,453 SNPs for F_ST_ and linkage disequilibrium (LD) analyses. Variation occurred mainly within populations (78.05%); 21.94% was among populations. F_ST_ was generally low (<0.05), indicating admixture, with a few sites showing higher differentiation (>0.15). Structure resolved three groups aligned with river’s upper, middle, and lower reaches. Rapid LD decay in LCH, LCS, and LJZ suggests recombination and moderate effective sizes. Results support conserving connectivity while protecting distinct units; LXZX and LWZ need targeted management.

**Abstract:**

This study examines the genetic diversity and population structure of silver carp (*Hypophthalmichthys molitrix*), an ecologically and economically important freshwater species. Samples were collected from 17 sites along the Yangtze River, including LCH, LCS, LJHK, and LXZX, as well as one population from the United States (SV). Restriction-site associated DNA sequencing (RAD-seq) generated 759,453 high-quality single-nucleotide polymorphisms (SNPs) for population genomic analyses, including genetic differentiation (F_ST_), population structure, and linkage disequilibrium (LD) decay. Genetic variation was primarily found within populations (78.05%), with 21.94% among populations. Most sites exhibited low genetic differentiation (F_ST_ < 0.05), suggesting high admixture along the river, although a few sites displayed elevated values (F_ST_ > 0.15). Rapid LD decay in LCH, LCS, and LJZ indicated frequent recombination and moderate to large effective population sizes. These patterns reflect the influence of geographic and ecological factors on population structure. Conservation strategies should maintain genetic connectivity while protecting distinct genetic resources. Populations with high differentiation, such as LXZX and LWZ, warrant targeted management to preserve unique genetic diversity.

## 1. Introduction

Silver carp (*Hypophthalmichthys molitrix*) is a plankton-feeding freshwater fish native to East Asia. It is taxonomically classified within the order Cypriniformes, family Cyprinidae, and genus Hypophthalmichthys, and ranks among China’s ‘Four Major Chinese Carps’ [1,2], an aquaculture designation, not a taxonomic rank, comprising black, grass, silver, and bighead carp. Silver carp is an efficient filter feeder that converts low-trophic plankton into biomass, enabling high-yield polyculture with bighead carp through niche partitioning. Its cultural importance, broad environmental tolerance, high fecundity, and reliable induced spawning underpin established hatchery systems and sustained demand. By regulating phytoplankton and recycling nutrients, it supports water quality management in ponds and integrated rice–fish systems, distinguishing this group from other farmed fishes outside China’s classical polyculture model [1,2]. This species holds significant economic importance in Asian aquaculture. The FAO dataset demonstrated that the annual production of silver carps exceeds 5.1 million metric tons worldwide at 2022 [3]. Its cost-effectiveness makes silver carp farming a crucial industry for food security, especially in low- to middle-income regions [4]. Notably, China dominates global production, contributing 3.91 million metric tons in 2024, which represented over 77.90% of worldwide silver carp output [5]. Paleontological and molecular phylogenetic analyses demonstrate bighead and silver carps originated from the Yangtze–Huanghe River basins, and modern populations may have derived from the secondary contact of geographically isolated fish during the last glacial events [6]. The native range of silver carp extends from approximately 20° N to 54° N, including the areas from the Red River (Northern Vietnam), Zhujiang (Pearl) River (southern China), and north to the Heilongjiang (Amur) River (the China–Russia border) [7,8].

The Yangtze River sustains diverse fish populations due to its varied topography, geomorphology, and climate [9]. In the Yangtze River, the middle stretches are critical for silver carp spawning [10]. However, wild silver carp populations have declined in recent decades, leading to the introduction of a 10-year fishing ban in 2020 [11]. Overfishing is a primary driver of this decline [12]. It reduces broodstock and juvenile populations, causing fish communities to become smaller and younger [13]. Large-scale water conservancy projects such as the Glen Canyon Dam project further fragment habitats, hindering fish migration and damaging spawning and nursery sites [14]. Land reclamation and water pollution also degrade habitats, hastening the loss of fish resources [11].

Genetic diversity is a fundamental concept in evolutionary biology and molecular ecology. It influences organismal complexity, ecosystem resilience, and a species’ capacity to adapt to diverse environmental conditions [15,16]. Studying adaptive divergence in relation to gene flow patterns enhances our understanding of speciation genomics [17]. Advantageous genetic diversity can be preserved under various selective regimes [18]. Genetic loci with elevated diversity maintained by balancing selection are particularly important for the survival of introduced populations adapting to new environments [18]. Identifying these loci and characterizing the associated genes involved in local adaptation can guide the development of strategies for managing biological invasions and reducing the impacts of non-native species [19]. Furthermore, determining the source populations and introduction routes of invasive species provides insights into the evolutionary forces shaping adaptation in response to environmental factors [19]. Such knowledge is valuable for optimizing the use of introduced resources in breeding programs and for implementing effective biological control measures [20].

To develop effective breeding programs and guide restocking and stock enhancement, it is essential to investigate the genetic background of *H. molitrix* populations. Previous studies have primarily assessed genetic variation using morphological traits [21], mitochondrial DNA [8,22], or microsatellites [23]. However, the population genetic structure of *H. molitrix* in the Yangtze River is still insufficiently understood, largely due to the limited availability of molecular markers (including simple sequence repeat, SSR; single-nucleotide polymorphisms, SNPs; amplified fragment length polymorphism, AFLP, and restriction fragment length polymorphism, RFLP) and small sample sizes in earlier research. Here, we employed RAD-seq to investigate the population genetic structure of silver carp in the Yangtze River. SNPs are identified and used to estimate genetic diversity, detect population differentiation, and infer historical population dynamics. This study offers a genomic perspective on the management and utilization of silver carp natural resources.

## 2. Materials and Methods

### 2.1. Ethical Statement

All experimental procedures were approved by the Laboratory Animal Welfare and Ethical Review, Institute of Hydroecology (IHE), Ministry of Water Resources and Chinese Academy of Sciences with Ethical Approval No.: IHE_IACUC_20170428_02 (Approval date: 28 April 2017).

### 2.2. Sampling Site and Methods

**Figure 1:**
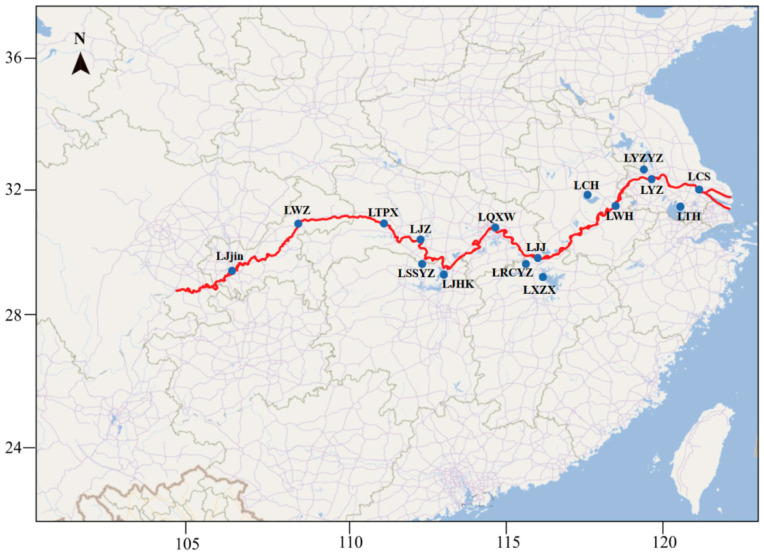
Sampling sites are shown on the river system map of the Yangtze River (Refer Table 1 for the full name of sampling site).

The Yangtze River Basin traditionally comprises the geographically starting and ending locations of each reach (upper, middle, and lower) based on geographic and hydrological features. The sampling point selection primarily relies on the natural distribution patterns of silver carp in the Yangtze River. The river is segmented into distinct sections by Yichang, Hubei Province. Upstream of Yichang, the river is impeded by the Three Gorges Dam and Gezhouba, creating unique hydrological and ecological conditions. Downstream, the river remains unobstructed, fostering better river connectivity. Sampling points were strategically placed in blocked upper reaches and natural river areas in the middle and lower reaches to capture the diverse distribution and correlation features of silver carp across habitats. As the river flows downstream, its current shifts from swift to gentle, accompanied by increasing anthropogenic activity. In 2017, we collected silver carp from 17 locations across the Yangtze (Figure 1), along with an additional site from the Marseilles Reach of the Illinois River (The Illinois River, a tributary of the Mississippi River, is linked to the Great Lakes via an artificial waterway that serves as both a corridor and a barrier against the spread of silver and bighead carp. Originally introduced to southern U.S. aquaculture systems in the 1970s for eutrophication control, both species escaped during major floods in the 1990s and rapidly colonized the Mississippi River and its tributaries. Their high fecundity, efficient plankton feeding, and lack of natural predators have enabled explosive population growth, severely disrupting native ecosystems. Despite eradication efforts since 2009, they continue to advance toward the Great Lakes, exemplifying the profound ecological risks posed by invasive alien species). Adult silver carp (1–2 kg) were captured using nets and identified by distinct morphological characteristics, such as the length of head over 30% of total body length with presence of an abdominal keel elongating from ventral fin to anus. The clips of caudal fin were taken from each specimen, fixed in 95% ethanol solution, and kept at –20 °C. Following tissue collection, all fish were released. In total, 181 specimens were collected. Summarized information of sampling is provided in Table 1.

### 2.3. Library Preparation, Construction, and Sequencing of RAD-Seq

Genomic DNA was isolated from each silver carp specimen via a commercial kit (Tiangen, Beijing, China) in accordance with the manufacturer’s instructions. The sample DNA qualities were assessed using 0.80% agarose gel electrophoresis and a spectrophotometer (ThermoFisher Scientific, Waltham, MA, USA). Concentrations of isolated DNA were measured with a Qubit 2.0 fluorometer (Life Technologies, Frederick, MD, USA). The isolated DNA with superior grade was then ready for library construction of RAD-seq at Frasergen Bioinformatics Co., Ltd. (Wuhan, China). Briefly, 500–1000 ng of isolated genomic DNA per individual was digested with the EcoRI, and the resulting fragments were then ligated to the Illumina P1 adapter (Illumina Inc., San Diego, CA, USA). Samples were pooled, ultrasonically sheared, and size-selected (300–700 bp) via agarose gel electrophoresis. The Illumina P2 adapter was then added, followed by PCR amplification to enrich for adapter-ligated fragments. Libraries were then pooled based on concentration and data requirements, and final RAD-sequencing was conducted on an Illumina HiSeq2000 platform (Illumina Inc., San Diego, CA, USA) with the parameter of 150 bp paired-end reads.

### 2.4. The Genotyping of Silver Carp

Raw reads were processed using fastp software (v0.18.0) [24] to trim those bases with a Phred quality score below 20, and to discard reads containing adapters or shorter than 50 bp. Clean reads were aligned with silver carp reference genome (HypMol1.0, Gen-Bank: GCA_037950675.1) using BWA-MEM (v0.7.17) [25]. Alignments were filtered with Samtools software (v1.90), and duplicates of PCR were deleted with the MarkDuplicates function in Picard Tools software (v2.13.2) (http://broadinstitute.github.io/picard/, accessed 17 September 2024). SNPs were called for each specimen using GATK Haplo-typeCaller software (v4.1.4.1) [26], followed by filtering with GATK VariantFiltration using the following thresholds: QD (QualByDepth) < 2.00, FS (FisherStrand) > 60.00, MQ (Mapping Quality) < 40.00, MQRankSum < –12.50, and ReadPosRankSum < –8.00. Filtered SNPs were combined per sample using GATK CombineGVCFs (default settings). Additional population-level filtering excluded variants with minor allele frequency < 0.01, missing genotypes in >20% of samples, or sequencing depth less than 4. The final high-confidence SNPs were taken for downstream population genetic analyses.

### 2.5. Linkage Disequilibrium Analysis and Genetic Diversity Assessment

Genetic parameters such as nucleotide diversity (Pi), observed heterozygosity (HO), expected heterozygosity (HE), and inbreeding coefficient (FIS) were assessed via VCFtools software (v0.1.13) [27]. Pi was estimated with a 5 kb sliding-window method. The Hardy–Weinberg equilibrium (HWE, minimum sequencing depth of 4 and a test threshold of 1 × 10^−6^) assessment was also conducted for each population in VCFtools. In addition, PopLDdecay software (v3.40) [28] was employed to assess linkage disequilibrium (LD) according to the filtered SNP dataset.

### 2.6. Structure Assessment and Population Differentiation Analysis

Pairwise F-statistics (F_ST_) were calculated using the software VCFtools (v0.1.13), employing the Weir and Cockerham method to assess genetic differentiation among all sampled populations [27]. The fixation coefficient (F) reflects the degree of allele heterozygosity within a population and represents a specific form of the F-statistic, commonly referred to as F_ST_. Specifically, *θ* denotes Weir and Cockerham’s estimator of F_ST_, which quantifies genetic differentiation among populations, the formula for the calculation was shown in Figure S1. We also conducted a nonparametric multivariate analysis of variance (non-parametric AMOVA) employing the adonis function in the Vegan package [29]. Population structure was analyzed using ADMIXTURE v1.3.0 [30], which employs a Bayesian clustering model based on the identified SNP markers. To explore the potential number of genetic groups (k), values ranging from 1 to 18 were tested. ADMIXTURE assigns individuals to specific genetic clusters and estimates the most likely population structure by minimizing the cross-validation error. The optimal k value is determined as the one with the lowest cross-validation error, representing the best-supported number of genetic clusters within the population. Ten independent runs were performed for each k. The value of k with the lowest cross-validation (CV) error was selected as the most likely representation of population structure.

A neighbor-joining phylogenetic tree (NJ) diagram was created via TreeBest software (v1.9.2) [31]. After SNP detection, the identified individual SNPs were used to calculate genetic distances between populations, from which a distance matrix was generated. This matrix served as the basis for constructing the NJ tree, providing insights into the evolutionary relationships among the populations. We conducted 1000 bootstrap replicates to assess tree reliability. The resulting NJ phylogenetic tree was visualized via iTOL (https://itol.embl.de, accessed on 17 September 2024). The principal components analysis (PCA) was carried out with GCTA (v1.91.4) [32]. We plotted the first two principal components using the ‘ggplot2’ package in R (v 4.0.0) [33]. The Markovian Coalescent model (MSMC) [34] was subsequently employed to estimate effective population size from genome-wide heterozygous sites. The Multiple Sequentially Markovian Coalescent (MSMC) model estimates the inverse coalescence rate (λ(t)), which was converted into effective population size (Ne) over time using the following formula: Ne(t) = 1/2 × λ(t) × μ, where λ(t) is the time-dependent coalescence rate and μ is the per-site, per-generation mutation rate.

## 3. Results

### 3.1. RAD-Sequencing and Statistic

RAD-seq generated 240 Gb of clean bases, yielding 834,893,237 clean reads. The average Q30 score was 97.76%, and the GC content was 39.57% which was very similar to the assembled genome (37.50%). Quality control statistics are presented in Table S1. On average, 96.19% of the sequencing data mapped to the reference genome. Of these mapped reads, 73.81% were properly mapped, while 0.49% were singletons (Table S2). Mean sequencing depth was 13.96×, and average genome coverage was 15.35% (Table S3). After high-quality filtering and SNP analysis, we identified 50,193,502 SNP loci. Of these, 28,489,173 were transitions (Ti) and 21,704,329 were transversions (Tv). The Ti/Tv ratio was 1.28 (Table S4). The total number of heterozygous was 34,764,000, while that of homozygous was 15,429,502.

### 3.2. Genetic Diversity Assessment

Genetic diversity metrics were estimated based on the identified SNPs. The in-breeding coefficient (FIS) ranged from −0.0719 in LYZ to 0.7691 in LWZ (Table S5). Mean nucleotide diversity (Pi) varied from 0.00029 (SV, LXZX and LYZYZ) to 0.0011 (LWZ). LXZX showed the lowest expected homozygosity (0.5826), while LWZ had the highest (0.9014). The LWZ showed the lowest expected heterozygosity (0.0985), while LXZX demonstrated the highest expected heterozygosity (0.4173). The number of SNPs deviating from Hardy–Weinberg equilibrium (HWE) ranged from 3607 in LJHK to 619,246 in LWZ. The total and final number of SNPs included in the population genetic analysis was 759,453.

### 3.3. Linkage Disequilibrium Analysis

**Figure 2:**
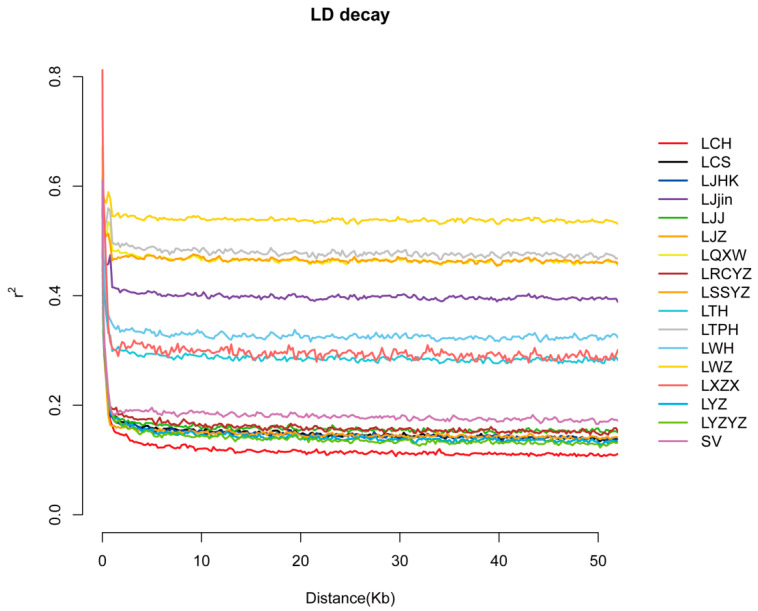
The linkage disequilibrium pattern decay pattern for the sampled silver carp population.

Based on the LD decay plot (Figure 2), most populations show a decrease pattern in LD (r^2^) within the first 10 kb. Their r^2^ values drop below 0.20 including LCH, LCS, LJHK, LJZ, LYZ, LSV, LRCYZ, LJJ, and LYZYZ and then plateau. In contrast, several populations (LQXW, LSSYZ, LTPH, and LWZ) maintain higher R^2^ values (about 0.50–0.60), reflecting a slower LD decay. This slower decay could result from smaller effective population sizes, historical bottlenecks, or restricted gene flow.

### 3.4. Genetic Differentiation Assessment

The nonparametric AMOVA (Table 2) shows that 21.94% of the genetic variation occurs among populations, while 78.06% is within populations. The F_model_ value of 2.8813 (*p* = 0.002) indicates significant differences among the populations. Several comparisons, such as LCH vs. LWZ (F_ST_ = 0.1814), LCS vs. LWZ (F_ST_ = 0.1629), LJHK vs. LWZ (F_ST_ = 0.1712), SV vs. LWZ (F_ST_ = 0.2059), and LXZX vs. LWZ (F_ST_ = 0.2177), display higher F_ST_ (above 0.15) (Table S6).

### 3.5. Population Structure Analysis

**Figure 3:**
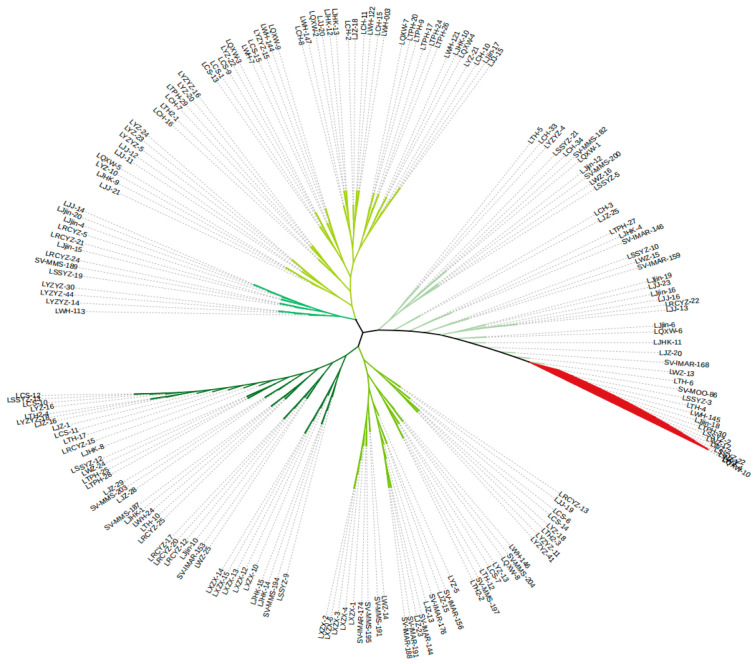
The phylogenetic tree analysis for the 181 silver carp individuals.

**Figure 4:**
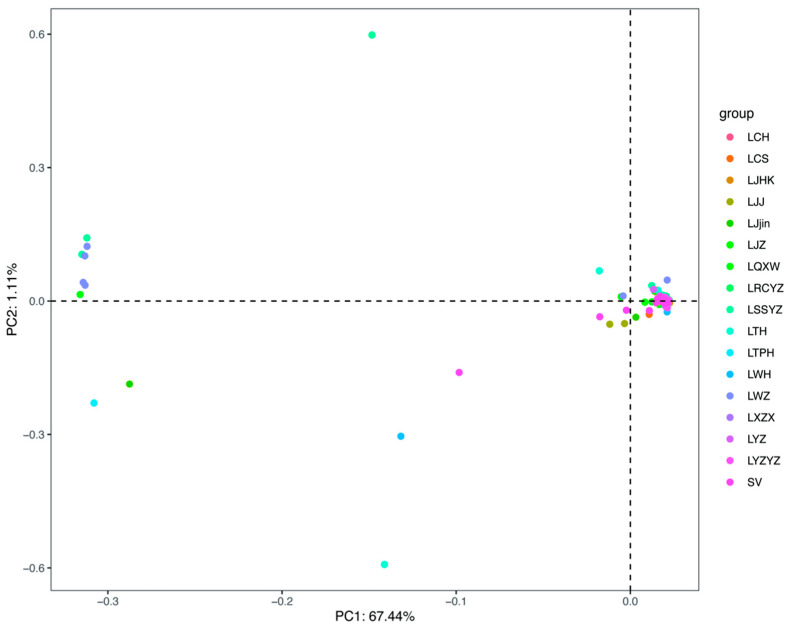
The PCA plot analysis with the first and second eigenvector for silver carp specimens.

We analyzed the population structure of 181 silver carp to clarify their genetic relationships. A phylogenetic tree (Figure 3) divided all samples into two distinct clades. Branch 1 contained 13 individuals from SV (1 individuals), LSSYZ (3 individuals), LTH (1 individuals), LWH (1 individuals), LJjin (1 individuals), LTPH (1 individuals), LWZ (4 individuals), and LQXW (1 individuals). The remaining 168 specimens grouped in Branch 2, which spanned multiple geographic branches. PCA supported these findings. PC1 explained 67.44% of the total variance, while PC2 ac-counted for 1.11% (Figure 4).

**Figure 5:**
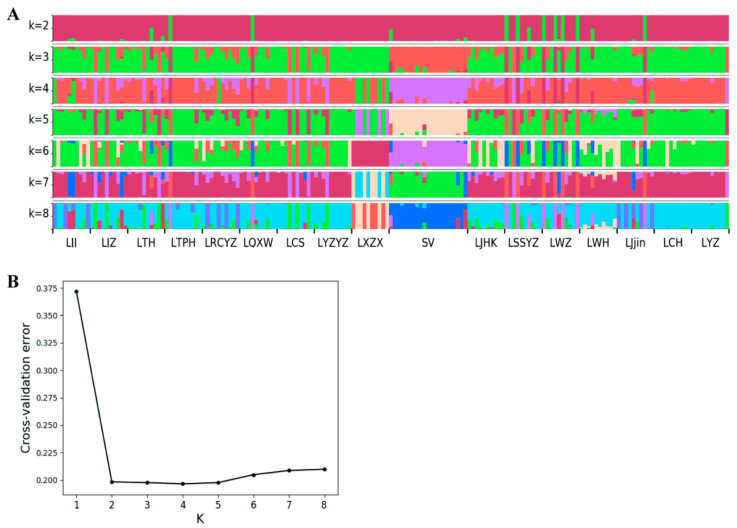
The population structure analysis of the sampled silver carp populations. (**A**) The admixture analysis. The length of each colored segment represents the proportion of the individual genome inferred from ancestral populations (k = 2~8). (**B**) Cross-validation (CV) error for different k values in admixture analysis, k = 4 is the optimal value.

Structure analysis revealed three main genetic groups among the 17 silver carp populations. One group consisted of most individuals from the Yangtze River Basin. Another included most SV individuals and a small portion from other Yangtze River populations. A third comprised nearly half of the LXZX individuals, plus smaller subsets from LJjin, LRCYZ, and LJJ. When k = 2, most Yangtze River Basin and United States populations clustered together, except for a few LSSYZ, LWZ, LWH, LJjin, LQXW, and LTH individuals (Figure 5A). At k = 3, most SV individuals grouped with some Yangtze River samples, while most Yangtze River samples and a few SV individuals formed another cluster. A third cluster encompassed some LTH, LQXW, LTPX, LSSYZ, LWZ, LWH, and LJjin individuals. When k = 4, nearly half of the LXZX individuals, along with smaller subsets of LJjin, LRCYZ, and LJJ, formed a distinct cluster. This approach assigned individuals to specific clusters, clarifying population structure. Cross-validation error indicated that k = 4 was optimal (Figure 5B).

### 3.6. Population Demographic History Assessment

**Figure 6:**
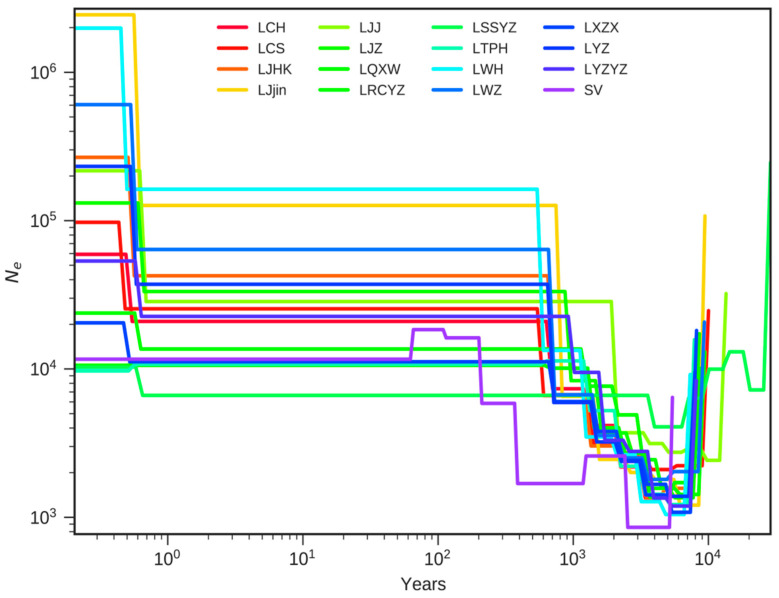
The estimated effective population size over time of the silver carp populations using the MSMC model.

Over time, many lines declined significantly, likely reflecting historical bottlenecks or environmental shifts. By around 10^2^–10^3^ years ago, most populations had reached lower effective sizes (10^3^–10^4^), although the timing and severity varied among groups (Figure 6). Some populations start near 10^5^–10^6^, then drop to about 10^4^ and hover there. The SV population shows a notable dip and partial rebound within the past 1000 years. Other lines mirror this high-to-moderate decline but at a more gradual pace, lingering in the 10^4^–10^5^ range for much of the timeline. The LWZ population also starts around 10^5^–10^6^, declines steadily, and levels off near 10^4^ with mild rebounds. In certain cases, populations remain above 10^5^ for extended periods before converging at 10^4^–10^5^. Overall, each population shows a broad pattern of high ancestral sizes followed by a pronounced bottleneck around 10^2^–10^3^ years ago. Minor differences in the timing and severity of these declines suggest that local factors—such as river-basin changes, historical fishing pressures, or stocking efforts—shaped each population’s demographic history.

## 4. Discussion

With the continual advancement of high-throughput sequencing technologies, fish population genetics research has become increasingly critical for elucidating key biological questions in ecology, evolutionary biology, and conservation science [35,36]. The capacity to generate large-scale genomic data rapidly and cost-effectively has revolutionized our ability to detect genetic diversity, track population structure, and infer evolutionary histories in both model and non-model fish species [37]. Moreover, these refined genetic insights have direct implications for assessing population viability under environmental change and anthropogenic pressures, thereby informing evidence-based conservation strategies to safeguard fish diversity and ecosystem health. In this study, the transition/transversion (Ti/Tv) ratio for silver carp (*H. molitrix*) was 1.28. Comparable RAD-seq estimates in other teleosts include *Hapalogenys analis* (1.59–1.87) [38], *Hemiculter leucisculus* (0.98) [39], *H. nobilis* (1.235) [2], *Esox masquinongy* (1.09–1.29) [40], and *Coilia nasus* (1.27) [41]. Collectively, these values place our estimate toward the lower end of the RAD-seq range reported for fish but within previously observed bounds. Variation among studies likely reflects enzyme choice, mapping strategy, and SNP-filtering thresholds.

LD is the non-random association of alleles at different SNP loci within a population. It is widely used to investigate evolutionary patterns and demographic processes [42]. Typically, the rate of LD decay in a population is positively correlated with its genetic diversity [2]. Faster LD decay often reflects larger effective population sizes and higher levels of recombination, while slower LD decay may indicate population bottlenecks or restricted gene flow [43]. Consequently, LD patterns can offer valuable insights into population history and genetic structure. Most populations in this study exhibit a sharp decline in linkage disequilibrium (LD) (r^2^) within the first 10 kb. Specifically, r^2^ values for populations such as LCH, LCS, LJHK, LJZ, LYZ, LSV, LRCYZ, LJJ, and LYZYZ drop below 0.2 before plateauing. This pattern reflects frequent re-combination events, which are indicative of moderate to large effective population sizes.

Rapid LD decay is often associated with healthy levels of genetic exchange within populations, driven by high recombination rates and sufficient genetic diversity. This trend can also suggest a stable demographic history, characterized by minimal effects from population bottlenecks or inbreeding. In comparison, species or populations with reduced effective sizes or constrained gene flow typically display slower LD decay due to limited recombination opportunities and elevated genetic drift [44]. These findings align with LD patterns reported in other fish species with high genetic diversity and large effective population sizes. Understanding LD decay patterns provides critical insights into the genetic structure, evolutionary history, and reproductive strategies of populations. The reproductive strategies of silver carp have evolved through long-term natural selection, enabling them to effectively adjust to shifts in freshwater ecosystems. Mass spawning enhances population sustainability by migrating to designated breeding grounds and reproducing at optimal times. This behavior creates a secure and plentiful habitat for eggs and juveniles, thereby guaranteeing the proliferation and growth of the silver carp population in dynamic natural settings. These insights are instrumental in guiding conservation and management strategies, particularly for maintaining genetic diversity and ensuring population resilience in the face of environmental changes or anthropogenic pressures.

The structure analysis revealed three primary genetic clusters among the 17 silver carp populations, delineating distinct patterns of genetic differentiation consistent with separate management units (MUs). The first genetic cluster predominantly comprised individuals from the Yangtze River Basin populations, reflecting a dominant genetic signature within this region. The second cluster included most individuals from the SV population along with a subset from other Yangtze River populations, suggesting shared ancestry or recent gene flow, likely indicating that the SV population originated from or was significantly influenced by Yangtze River populations. The third genetic cluster consisted primarily of nearly half of the LXZX individuals and smaller subsets from LJjin, LRCYZ, and LJJ populations, indicative of localized genetic divergence. At k = 2, populations from the Yangtze River Basin and the introduced U.S. populations largely clustered together, with minor sub-structuring observed in populations such as LSSYZ, LWZ, LWH, LJjin, LQXW, and LTH. Increasing the resolution to k = 3 resulted in the partial separation of the SV population, while an additional cluster emerged encompassing populations including LTH, LQXW, LTPX, LSSYZ, LWZ, LWH, and LJjin. At k = 4, the LXZX population, along with subsets from LJjin, LRCYZ, and LJJ, formed a distinct cluster, highlighting fine-scale genetic structure. The observed genetic structure in silver carp parallels findings in other freshwater fish species. For example, bighead carp and grass carp also display genetic clustering influenced by geographic barriers and ecological conditions [6,45,46]. Additionally, silver carp’s genetic structure reflects patterns in riverine species such as black carp, where habitat fragmentation and human activity influence genetic diversity and population connectivity [47].

The findings highlight the importance of preserving population-specific genetic diversity in silver carp. Local population, such as those in LXZX and subsets of LJjin, LRCYZ, and LJJ, are particularly vulnerable to genetic erosion due to limited connectivity. Conservation strategies should aim to maintain genetic exchange among populations while protecting unique genetic resources [48]. Comparisons with other species under-score the critical role of geographic and ecological factors in shaping genetic structure and inform broader strategies for managing freshwater fish populations. Understanding genetic relationships between native and introduced populations can guide management to mitigate ecological impacts while maintaining native genetic integrity. Overall, the observed genetic structure provides valuable insights into the evolutionary history and demographic dynamics of silver carp, with direct applications to conservation and resource management.

Most pairwise F_ST_ values are low (below 0.05), suggesting minimal differentiation and recent or ongoing gene flow. However, higher F_ST_ values (>0.15) in comparisons involving LWZ such as LCH vs. LWZ, F_ST_ = 0.1814; SV vs. LWZ, F_ST_ = 0.2059; LXZX vs. LWZ, F_ST_ = 0.2177 indicate strong population subdivision and limited connectivity. Such differences may arise from historical isolation, habitat fragmentation, or local adaptations. Overall, the range of F_ST_ values indicates varying levels of connectivity, with some groups closely related and others showing greater divergence. The low overall differentiation with pockets of high divergence is consistent with other fish species. For instance, grass carp and bighead carp show low F_ST_ across broad ranges, with occasional elevated values tied to geographic or ecological barriers [45,46]. Similarly, riverine species like carp species display localized genetic differentiation influenced by habitat fragmentation [49,50]. For silver carp, the findings highlight a balance between connectivity and localized genetic distinctiveness. In the study area, the Yangtze River is obstructed by the Three Gorges Dam and the Gezhouba Dam—two major hydrological barriers located approximately 38 km apart at the junction between the upper and middle reaches of the river. These structures disrupt natural fish migration routes and impede genetic exchange between silver carp populations in the upper Yangtze and those in the middle and lower reaches [51,52].

Conservation strategies should aim to preserve connectivity to maintain genetic diversity while protecting unique populations like LWZ, which may harbor important adaptations. This dual approach is crucial for ensuring long-term resilience, similar to management strategies for other freshwater species with complex population structures. We proposed that protecting populations with high diversity is a priority, particularly those of silver carp with rich genetic diversity, characterized by a high number of alleles and heterozygosity. These populations are crucial for the evolutionary potential of the species. For populations in the middle and lower reaches of the Yangtze River with good natural connectivity and frequent gene exchange, safeguarding their spawning grounds, limiting water area development, preserving habitats such as shoals, aquatic grass areas, maintaining food webs and breeding grounds are essential to provide a stable evolutionary foundation. Regular genetic monitoring is necessary for populations with low genetic diversity. If heterozygosity declines and inbreeding signals emerge, timely ecological regulation, artificial propagation, or release interventions should be implemented to slow genetic degradation. Isolated populations, like LWZ, require special management, involving analysis of their genetic uniqueness like unique alleles, adaptive mutations through whole genome sequencing. If these genetic resources hold potential for the species to adapt to future environmental changes, they are classified as genetic protection units, subject to stricter protection to preserve unique evolutionary lineages.

## 5. Conclusions

Utilizing 759,453 RAD-seq SNPs, our analysis reveals that silver carp populations in the Yangtze River harbor the majority of their genetic diversity within populations (78.05%). Overall, genetic differentiation is minimal (F_ST_ < 0.05), suggesting significant gene flow along the river. However, a small number of sites exhibit notable divergence (F_ST_ > 0.15). Population stratification identifies three distinct groups corresponding to the upper, middle, and lower regions of the river. Specifically, LCH, LCS, and LJZ populations demonstrate rapid decay of linkage disequilibrium, indicating frequent recombination events and substantial effective population sizes.

The findings endorse a dual conservation strategy: one, to uphold connectivity for gene flow and resilience, and two, to safeguard distinct units like LXZX and LWZ to preserve exclusive genetic reservoirs. This equilibrium approach will aid in safeguarding existing genetic diversity and region-specific adaptations amidst environmental shifts. The dataset sets a genomic foundation for conservation strategies, encompassing surveillance, hatchery protocols, and potential relocations. Subsequent research should integrate genomic information with environmental variations and temporal samplings to elucidate factors influencing differentiation and to monitor population dynamics over time.

## Figures and Tables

**Table 1:** The statistic information of the 17-sampling site of silver carp population.

Population ID	Sampling Site	River System	Sample Size
LJjin	Jiangjin, Chongqing, China	Upper reach of the Yangtze River	10
LWZ	Wanzhou, Chongqing, China		10
LTPH	Taipingxi, Hubei, China		10
LJZ	Jingzhou, Hubei, China	Middle reach of the Yangtze River	10
LQXW	Qixingwan, Hubei, China		10
LJJ	Jiujiang, Jiangxi, China		10
LWH	Wuhu, Anhui, China	Lower reach of the Yangtze River	10
LYZ	Yangzhou, Jiangsu, China		10
LCS	Changsu, Jiangsu, China		10
LJHK	Dongting lake, Hunan, Chian	Major Yangtze-connected Lakes in the middle and lower reaches of the Yangtze River	10
LXZX	Poyang lake, Jiangxi, China		10
LCH	Chaohu lake, Anhui, China		10
LTH	Taihu lake, Jiangsu, China		10
LYZYZ	Yangzhou Hatchery, Jiangsu, China	The broodstock of a national hatchery in the middle and lower reaches of the Yangtze River	10
LRCYZ	Ruichang Hatchery, Jiangxi, China		10
LSSYZ	Shishou Hatchery, Hubei, China		10
SV	The Marseilles Reach of the Illinois River	Mississippi River	21

**Table 2:** The statistic of the nonparametric AMOVA of the sampled silver carp populations.

Source of Variation	d.f.	Sum of Squares	MeanSqs	F_model_	R^2^	Pr (>F)
Among populations	16	148.9617	9.3101	2.8813	21.94%	0.002
Within populations	164	529.9067	3.2311		78.06%	
Total	180	678.8684				

## Data Availability

Dataset available on request from the authors.

## Supplementary Materials

The following supporting information can be downloaded at: https://www.mdpi.com/article/10.3390/ani15192906/s1, Figure S1: Formula for quantifies genetic differentiation among populations; Table S1: QC statistic; Table S2: Mapped statistic; Table S3: Sequencing depth statistic; Table S4: SNP statistic; Table S5: Genetic diversity statistic; Table S6: F-statistic (FST).
