# Supplementary figures and images for: Population Genomic Survey of Hypophthalmichthys molitrix in the Yangtze River Basin: A RAD Sequencing Perspective
Source: Animals (Basel). 2025 Oct 5;15(19):2906. doi: 10.3390/ani15192906 (PMC12524325; doi:10.3390/ani15192906)

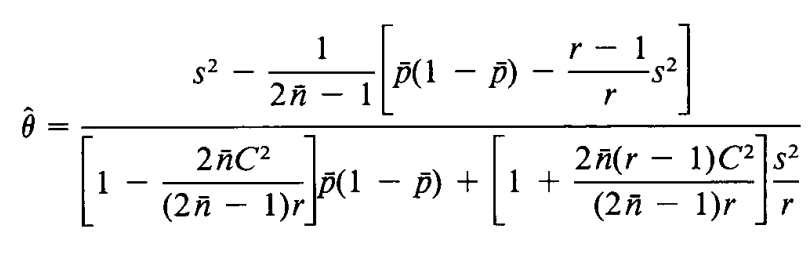

Supplement: Supplementary file 1 [file animals-15-02906-s001.zip › Figure S1.png]
